# Is It Her Swan Song?

**Published:** 1919-11-15

**Authors:** 


### IS IT HER SWAN SONG?

This question rose to the minds of some of the audience at the first meeting of an embryo body calling itself a Professional Union of Trained Nurses, and in regard to the lady who, for her own purposes, occupied a second-row seat in the hall where the embryo first meeting took place on October 25.

She appeared again at the second meeting at the Y.M.C.A. Hall, Tottenham Court Road, on the 6th inst., when those attending were less numerous; all members of the male sex, including the lawyers, had deserted or remained away, and it appeared that an overwhelming majority of the 150 or so present were out and out followers of the lady who described herself to the audience as the "old war horse and leader," and inferentially in the last issue of her print, the B. J. N., of the 8th, constituted "One of the Immortals and Great Nursing Pioneers in History." It is not impossible that our American friends may enjoy themselves on learning that when speakers ran short she took the floor, and roundly trounced the College of Nursing in language too terrible to admit to our prosaic pages. They may naturally inquire what justification is there for a trained nurse, who labels herself "the old war horse and leader, one of the immortals and great nursing pioneers in history," to set herself the task of encouraging poor nurses in need of work to place their destinies in the hands of "a Trades Union under the ordinary Trades Union rules and practice," to their manifest undoing and probably ultimate forfeiture of a successful professional life as trained nurses? It is true she did not, we believe, even mention the words "Trades Union," though she had brought apparently most of her supporters to the second meeting of representatives of the Professional Union of Trained Nurses, registered under the Trades Union Act.

Miss MacCallum was in the chair, and amongst other things she said that nurses were treated as "a cross between an angel and an automatic machine," but she did not produce the usual photographs of the product thus created, nor did she state where the resulting mule could be met with.

The best speech was made by a Miss Klaassen, of the National Union of Scientific Workers, a moderate, impartial and just speaker, the best of the bunch.

Miss Ferrier, a member of the College of Nursing, pointed out that during thirty years the Central Committee had spent £22,000, and had achieved nothing. She also pointed out that the College six months ago had issued a report which had helped to improve salaries, to lessen hours, to institute clubs, together with a loan fund. Nothing had been done by the Central Committee. In March of this year there had been a meeting at the Mansion House to found a club for the R.B.N.A., but where was the club? Where was the beginning of the club? Members of the R.B.N.A. had benefited by the much-abused Nation's Fund. This statement met with denials from the audience, one lady ejaculating the word "lies" several times over, adding emphasis to each as her temperature rose. Miss Macdonald laid the blame on State Registration, which blocked the way.

Mrs. Bedford Fenwick ignored altogether the objects of the meeting of the Professional Union of Trained Nurses. She gave a general denial to some of the statements of Miss Ferrier. She denounced the College of Nursing, root and branch, its 16,000 misguided nurse members, and marvelled that they had the courage to call themselves British women. She blamed the nurses because they did not manage their own affairs, accused them of mental and physical apathy, and blamed them for this, though she omitted to point out that she had once been a matron in charge of a training-school; but perhaps that fact had escaped her memory, as it was, we believe, some thirty years ago, and her knowledge of the modern training-school and system of training nurses must be imperfect to say the least. She referred to the money transactions mentioned by Miss Ferrier without throwing much light upon them, and stated that she had saved the nurses from the greatest danger to which any body of women had ever been subjected, for she had won them their freedom and led them to noble victory.

Miss Jessie Holmes, of the American Red Cross Maternity Hostel at Bermondsey, made an excellent little speech which was very welcome after the rude interruptions and noise of the organised followers of the self-named "old war horse and leader" whose polemics were applauded to the echo and received by the grateful recipient with cheerful waves of the hand. **Was all this an eclipse for "a professional trades union of nurses?"**

